# Using behavioural theories to optimise shared haemodialysis care: a qualitative intervention development study of patient and professional experience

**DOI:** 10.1186/1748-5908-8-118

**Published:** 2013-10-07

**Authors:** Liz Glidewell, Stephen Boocock, Kelvin Pine, Rebecca Campbell, Julia Hackett, Shamila Gill, Martin Wilkie

**Affiliations:** 1 Leeds Institute of Health Sciences, University of Leeds, Charles Thackrah Building, 101 Clarendon Road, Leeds, UK; 2 Yorkshire and the Humber Specialised Commissioning Group, NHS Barnsley, Hillder House, 49-51 Gawber Road, Barnsley, UK; 3 Sheffield Teaching Hospitals NHS Foundation Trust, Sheffield Kidney Institute, Northern General Hospital, Herries Road, Sheffield, UK

**Keywords:** Haemodialysis, Shared care, Behavioural theory, Barriers and facilitators

## Abstract

**Background:**

Patients in control of their own haemodialysis report better outcomes than those receiving professional controlled care in a hospital setting, even though home and hospital haemodialysis are largely equivalent from mechanical and physiological perspectives. Shared Haemodialysis Care (SHC) describes an initiative in which hospital haemodialysis patients are supported by dialysis staff to become as involved as they wish in their own care; and can improve patient safety, satisfaction and may reduce costs. We do not understand why interventions to support self-management in other conditions have variable effects or how to optimise the delivery of SHC. The purpose of this study was to identify perceived patient and professional (nurses and healthcare assistants) barriers to the uptake of SHC, and to use these data to identify intervention components to optimise care.

**Methods:**

Individual semi-structured interviews with patients and professionals were conducted to identify barriers and facilitators. Data were coded to behavioural theory to identify solutions. A national UK learning event with multiple stakeholders (patients, carers, commissioners and professionals) explored the salience of these barriers and the acceptability of solutions.

**Results:**

A complex intervention strategy was designed to optimise SHC for patients and professionals. Interviews were conducted with patients (n = 15) and professionals (n = 7) in two hospitals and three satellite units piloting SHC. Data from patient and professional interviews could be coded to behavioural theory. Analyses identified key barriers (knowledge, beliefs about capabilities, skills and environmental context and resources). An intervention strategy that focuses on providing, first, patients with information about the shared nature of care, how to read prescriptions and use machines, and second, providing professionals with skills and protected time to teach both professionals/patients, as well as providing continual review, may improve the implementation of SHC and be acceptable to stakeholders.

**Conclusions:**

We have developed an intervention strategy to improve the implementation of SHC for patients and professionals. While this intervention strategy has been systematically developed using behavioural theory, it should be rigorously tested in a subsequent effectiveness evaluation study prior to implementation to ensure that shared haemodialysis care can be delivered equitably, efficiently and safely for all patients.

## Background

Patients in control of their own haemodialysis report better outcomes than those receiving professional controlled care in a hospital setting [1-3]. Hospital haemodialysis occurs in a hospital or satellite centre where nursing or technical staff provide day-to-day dialysis care [4]. It has been characterised as rigid and inflexible with no or limited patient involvement [5,6]. Dialysis treatment is time consuming for patients; haemodialysis needs to be minimally disruptive and feasible in routine care. The prevalence of the dialysis population is rising at approximately 5% per annum given the ageing population and the rise of type 2 diabetes and cardiovascular disease [7]. The costs associated with managing end-stage renal disease are high in relation to the proportion of people affected, dialysis accounting for between 2% to 3% of healthcare budgets even though it affects 0.02% to 0.03% of the population in developed countries [8]. Hospital-based haemodialysis mortality rates remain extremely high (15% annually in the US) and associated morbidity (*e*.*g*., hypertension, cardiac sequelae, mineral and bone disorders, and psychological disorders) implies that delivery of haemodialysis is sub-optimal [4,9]. Poorer outcomes remain consistent even after adjusting for demographics and co-morbidities.

Shared Haemodialysis Care (SHC) describes an initiative in which centre-based haemodialysis patients are supported by dialysis staff to become as involved as they wish to in their own care. This approach can impact beneficially on several domains of healthcare, including patient-centredness, equity of access, patient safety, timeliness, effectiveness of treatment, and efficiency of resource use [10-12]. SHC differs from self-care in that the professional and individual share responsibility for the patient’s treatment, health and well-being [13], with the professional adopting a facilitator role rather than performing repetitive tasks to a passive patient. Patients become active in their care at a rate and level determined by themselves in partnership with healthcare professionals [1]. SHC aims to maximise functioning and well-being, and minimise the emotional impact of haemodialysis. People who actively participate in their own care are thought to reduce variation in care, as patients only get the care that they need and want [1]. SHC is considered to improve the role of the healthcare professionals, as time saved performing routine tasks can allow nurses to deal with more complex cases and spend more time educating and supporting patients in a holistic manner [5]. Patient and professional satisfaction is thought to be increased as the relationship is expanded to focus on the person, his or her life, and the patient’s other health problems [14]. Patient engagement in long term conditions where patients are fully activated (engaged and involved) with their own health is recognised as an important strategy for effective management of health resource use [15].

This study is nested within a larger quality improvement initiative that aims to transform hospital-based haemodialysis in one region of the UK from a staff-led service to a patient-staff shared self-management program (http://www.health.org.uk/areas-of-work/programmes/closing-the-gap-through-changing-relationships/related-projects/from-dependency-to-control-enabling-self-dialysis-in-hospital/). Shared haemodialysis care involves many tasks that range from relatively simple (*e*.*g*., recording weight) to more complex tasks that involve a series of inter-related tasks (*e*.*g*., self-needling the fistula) (see list of SHC competencies). In collaboration, patients and professionals (nurses and healthcare assistants) select which competencies to undertake [16]. Participating centre data indicate that the majority of haemodialysis patients are interested in shared-dialysis (90%, December 2012 unpublished Questionnaire Survey York and Sheffield Teaching Hospitals). However, only some UK patients currently have access to shared-dialysis, creating inequity that may result in inequalities in patient outcomes and service provision.

List of SHC competencies

1. Takes weight

2. Takes blood pressure and pulse

3. Takes temperature

4. Washes hands and arm

5. Lines machine

6. Primes machine

7. Prepares dressing pack ready for access

8. Programmes machine using prescription

9. Inserts one or both needles into AV Fistula/Graft or prepares tunnelled line for dialysis

10. Hooks up, bleeds out, and commences dialysis

11. Has completed 'Problem Solving Competency’ in Shared Care Handbook

12. Discontinues dialysis by hooking up and washing back

13. Presses needle sites after removal

14. Administers any injections via dialysis machine or subcutaneously

The UK Medical Research Council has produced guidance that called for the systematic development of complex interventions to change practice [17,18]. Evidence translated to patients, healthcare practitioners, local administrators, and national policy makers may be more likely to be implemented if informed by an assessment of barriers and facilitators. Social scientists have developed a practical theory-based framework [19,20] to systematically identify why implementation of best practice fails. The Theoretical Domains Framework synthesises psychological constructs from 33 theories relevant to understanding professionals’ implementation behaviour. A total of 17 published studies involving healthcare professionals and 4 studies involving members of the public have been published [21], illustrating its potential to understand behaviour in other clinical areas. This study aimed to explore whether the framework could further our understanding of barriers and facilitators of patient and professional behaviour.

We do not understand why interventions to support self-management have variable effects or how to optimise the delivery of SHC. SHC requires both professionals and patients to change their behaviour. Shared-care researchers have identified factors that prevent and enable its use in other contexts (*e*.*g*. professional attitudes, organisational culture, resources and time pressures in other conditions) [22,23]. These barriers can span individual, team, and system level factors, but current research has not incorporated these multiple perspectives as they have used a grounded approach to identifying benefits/barriers. In addition, the shared-care empirical evidence-base neglects the perspective of patients and professionals who are not general practitioners [21]. The purpose of this study was to identify perceived patient and professional (nurses and healthcare assistants) barriers to the uptake of SHC, using behavioural theory, and to use these data to identify intervention components to optimise care. A secondary aim was to explore whether the Theoretical Domains Framework developed to understand the implementation behaviour of professionals could be applied to patient implementation behaviour.

## Methods

Individual semi-structured interviews with patients and professionals were conducted to identify barriers. Data were coded to behavioural theory to identify solutions. A national UK learning event with multiple stakeholders (patients, carers, commissioners and professionals) explored the salience of these barriers and the acceptability of solutions.

### Aims

To understand how people with end-stage renal failure and the healthcare professionals involved in their care experience SHC. These data were coded to behavioural theory [19] to build a tailored intervention strategy that could improve the implementation of SHC for patients and professionals.

### Objectives

1. To understand the range of patient experience in different types of patient who: undertake SHC (all 14 competencies); undertake some SHC (up to 5 competencies); and opt out of SHC (see list of SHC competencies).

2. To understand the day-to-day healthcare professional experience of delivering SHC from the perspective of nurses and healthcare assistants.

3. To systematically identify barriers and facilitators to implement SHC.

4. To identify which intervention components may aid implementation of SHC that should be tested in a subsequent rigorous evaluation.

5. To explore whether the Theoretical Domains Framework (developed to understand the implementation behaviour of professionals) can be used to explore patient implementation behaviour.

### Study design

Individual semi-structured interviews were conducted with consenting patients and professionals to understand their retrospective experience of SHC. Open questions were used to elicit their personal experiences (*e*.*g*., 'What do you normally do when you attend for haemodialysis?’). The topic guide focussed on the personal experience, and theoretical prompts were informed by the Theoretical Domains Framework (*e*.*g*., knowledge, beliefs, etc.) (see Table 1, topic guide) [19,24]. The topic guide was piloted with three participants and revised to focus on the personal experience of haemodialysis. A national learning event of key stakeholders explored the salience of barriers and facilitators and appropriateness of the proposed implementation strategy.

**Table 1:** Topic guide

This illustrative interview schedule was adapted for patients and professionals.	Other prompts:
Participants will be asked a series of questions to elicit their perspectives. We will ask participants to talk about their experience of shared haemodialysis. Domains will include: beliefs about haemodialysis; experience of haemodialysis; and attitudes towards shared haemodialysis care.	• Knowledge about Shared Haemodialysis Care
Patients and professionals were asked similar questions (adapted for purpose):	• Recent vs. long term problems and benefits
Beliefs about haemodialysis.	• If thought about participating/opting out
Can you tell me how you came to be on haemodialysis?	• Any exit strategy
Prompts: what happened; how long ago; what for? (This will help ascertain trajectory).	• Self-care (as opposed to nurse-led care)
What do you usually do when you come for dialysis?	• Consequences (benefits/risks) of taking and how to balance this out
Experience of haemodialysis.	• What problems/concerns do you have about shared haemodialysis – both short-term and long-term
What effect does haemodialysis have on your life?	• Mood, functional status, QOL
Prompts: quality; what can do/can’t do; how are things different/the same?	• Support from others
Attitudes towards shared haemodialysis.	• If interview has raised any concerns and if these will be discussed with the named nurse
How you do you feel about participating in shared haemodialysis?	

### Participants

Patients who attend for haemodialysis in two hospital and three satellite centres piloting SHC in the Yorkshire and Humber region of the UK were invited by post to participate in a face-to-face semi-structured interview (lasting approximately one hour). The five centres were pragmatically selected, as they were the first to implement a training course for healthcare professionals in the UK. SHC involves a number of competencies (see list of SHC competencies), and all patient participants were categorised as: those undertaking all fourteen elements of SHC; those undertaking some SHC (up to five competencies); and those opting out of SHC (see list of SHC competencies).

Nurses and healthcare assistants involved in all, some, or opting out of SHC were identified by the matrons of each centre. Healthcare Assistants are members of the care team who provide direct patient care (*e*.*g*., Insertion of arterio-venous fistula needles/care of tunnelled lines; preparation and disconnection haemodialysis therapy according to prescription; and monitoring and observation of the patient and machine during automated haemodialysis) supervised by registered nurses. Only professionals involved in the day-to-day delivery of SHC were invited to participate; therefore, other team members such as dieticians, nephrologists, and social workers were excluded.

### Sample

**Figure 1:**
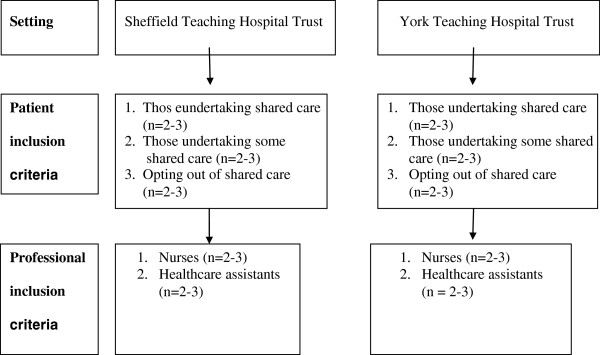
Participant involvement flowchart.

To provide insight into the experience of SHC, we planned to purposively recruit two to three participants from each of our inclusion criteria (level of involvement [opt out, some, all] and type of participant patient, nurse/healthcare assistant, see Figure 1 and Table 2, participant characteristics). Patients (involved in piloting SHC 256 on dialysis) and professionals (106 nurses and Healthcare Assistants) associated with two teaching hospital haemodialysis centres in Yorkshire and Humber UK were considered eligible.

**Table 2:** Participant characteristics

	**Patients invited**		**Patients interviewed**		**Total interviewed**
**Patient level of engagement**	**Sheffield**	**York**	**Sheffield**	**York**	
	**Total (male)**	**Total (male)**	**Total (male)**	**Total (male)**	
None	4 (3)	6 (2)	3 (2)	1 (1)	4
Some	1 (1)	9 (7)	1 (1)	5 (4)	6
Full	5 (3)*	1 (0)	4 (3)	1 (0)	5
**Total patients interviewed**	10 (7)	16 (9)	**8 (6)**	**7 (5)**	**15**
	**Staff invited**		**Staff interviewed**		**Total interviewed**
	**Sheffield**	**York**	**Sheffield**	**York**	
**Nurse level of engagement**					
None		1 (1)		1 (0)	
Some	2 (1)	1 (0)	2 (1)	1 (0)	
Full	2 (1)	1 (0)	2 (1)	0	
**Total nurses interviewed**	4 (2)	3 (1)	**4 (2)**	**2 (1)**	**6 (3)**
**Healthcare assistant level of engagement**					
None	0	1 (0)	0	0 (0)	
Some	0	1 (0)	0	1 (0)	
Full	0	0	0	0	
**Total healthcare assistants interviewed**	0	2 (0)	**0**	**1 (0)**	**1 (0)**

Note. Figures in brackets equal number of male participants.

* Female patient died between pack being mailed and interview time.

#26 patients contacted, 19 responded, 3 opted out (all female, not engaged in SHC).

#7 staff contacted, 7 responded, 0 opted out.

### Patient and professional recruitment

Nursing staff external to the project team at the respective centres familiar with these patients (and the level of shared haemodialysis undertaken by them) identified potential participants and mailed recruitment packs containing a letter of invite, participant information sheet, and stamped addressed envelope.

The matrons processed letters of invitation and mailed recruitment packs to professionals. Only participants who indicated that they wished to hear more about the project were made identifiable to the research team. No reminders were sent to non-responders. Individuals who replied that they would like to know more about the project were phoned by the research team. After the person had the opportunity to discuss the project, they were asked if they wished to take part, and a mutually convenient time to be interviewed was arranged.

Participants were given a £20 book voucher in recognition of their time. Anonymous information relating to level of SHC and gender was collected for descriptive purposes on participants who did not consent to take part. Participant demographic information was not collected to protect anonymity.

### Interview setting and conduct

Individual semi-structured interviews with patients and professionals were conducted at a time and place convenient to them. Patients were interviewed by either a patient (SB), a former caregiver (KP), or an implementation researcher (LG); all professional interviews were conducted by LG. All interviews were digitally recorded.

### Analysis

Transcripts were read and re-read to familiarise the research team (LG, SB, KP and JH) with the data. Data were coded to theoretical constructs (see Table 3) and emerging themes within these constructs by LG. The reliability of coding was explored between two researchers (LG and JH) on a sample of data. One transcript was divided into meaning units (sentences or utterances), and inter-rater coding reliability was explored qualitatively given the number of constructs (n = 14). We did not undertake participant validation.

**Table 3:** **Theoretical domains**[24]

**Domain (definition)**	**Constructs included**
Knowledge (An awareness of the existence of something)	Knowledge (including knowledge of condition/scientific rationale)
	Procedural knowledge
	Knowledge of task environment
Skills (An ability or proficiency acquired through practice)	Skills
	Skills development
	Competence
	Ability
	Interpersonal skills
	Practice
	Skill assessment
Social/Professional Role and Identity (A coherent set of behaviours and displayed personal qualities of an individual in a social or work setting)	Professional identity
	Professional role
	Social identity
	Identity
	Professional boundaries
	Professional confidence
	Group identity
	Leadership
	Organisational commitment
Beliefs about Capabilities (Acceptance of the truth, reality, or validity about an ability, talent, or facility that a person can put to constructive use)	Self-confidence
	Perceived competence
	Self-efficacy
	Perceived behavioural control
	Beliefs
	Self-esteem
	Empowerment
	Professional confidence
Optimism (The confidence that things will happen for the best or that desired goals will be attained)	Optimism
	Pessimism
	Unrealistic optimism
	Identity
Beliefs about Consequences (Acceptance of the truth, reality, or validity about outcomes of a behaviour in a given situation)	Beliefs
	Outcome expectancies
	Characteristics of outcome expectancies
	Anticipated regret
	Consequents
Reinforcement (Increasing the probability of a response by arranging a dependent relationship, or contingency, between the response and a given stimulus)	Rewards (proximal / distal, valued / not valued, probable / improbable)
	Incentives
	Punishment
	Consequents
	Reinforcement
	Contingencies
	Sanctions
Intentions (A conscious decision to perform a behaviour or a resolve to act in a certain way)	Stability of intentions
	Stages of change model
	Transtheoretical model and stages of change
Goals (Mental representations of outcomes or end states that an individual wants to achieve)	Goals (distal / proximal)
	Goal priority
	Goal / target setting
	Goals (autonomous / controlled)
	Action planning
	Implementation intention
Memory, Attention and Decision Processes (The ability to retain information, focus selectively on aspects of the environment and choose between two or more alternatives)	Memory
	Attention
	Attention control
	Decision making
	Cognitive overload / tiredness
Environmental Context and Resources (Any circumstance of a person’s situation or environment that discourages or encourages the development of skills and abilities, independence, social competence, and adaptive behaviour)	Environmental stressors
	Resources / material resources
	Organisational culture /climate
	Salient events / critical incidents
	Person x environment interaction
	Barriers and facilitators
Social influences (Those interpersonal processes that can cause individuals to change their thoughts, feelings, or behaviours)	Social pressure
	Social norms
	Group conformity
	Social comparisons
	Group norms
	Social support
	Power
	Intergroup conflict
	Alienation
	Group identity
	Modelling
Emotion (A complex reaction pattern, involving experiential, behavioural, and physiological elements, by which the individual attempts to deal with a personally significant matter or event)	Fear
	Anxiety
	Affect
	Stress
	Depression
	Positive / negative affect
	Burn-out
Behavioural Regulation (Anything aimed at managing or changing objectively observed or measured actions)	Self-monitoring
	Breaking habit
	Action planning

*Note*. All definitions are based on definitions from the American Psychological Associations’ Dictionary of Psychology.

Analysis involved two stages-where distinct themes were apparent, data were coded deductively to constructs of the Theoretical Domains Framework; additional inductive themes not captured were looked for by LG. Verbatim coded data arising from the analysis were plotted using the framework methodology to theoretical domains (see Table 3) to consider the content of the data elicited from the different categories of: patient undertaking all SHC; undertaking some SHC; and opted out of SHC; and healthcare professional: nurse and healthcare assistant. Disconfirming and deviant cases were looked for by the research team. A matrix developed to build interventions [25] was used along with the expertise of the research team (clinicians, professionals, clinician educators, commissioners) and feedback from a shared learning event to explore the salience of barriers/facilitators and explore the acceptability of proposed intervention strategies. Stakeholders attending the learning event included patients, caregivers, nurses, consultants, and policy makers.

## Results

A complex intervention strategy was designed to optimise SHC for patients and professionals. Verbatim quotes are presented by key barrier/enablers in Tables 4 and 5. Where relevant quotes are cited, they are annotated with the construct (*e*.*g*. knowledge or skills), setting (Sheffield or York), order of interview, and followed by the participant (patient or professional) in the table. Results for patients and professionals are grouped in separate tables (see Tables 4 and 5).

**Table 4:** Patient barriers and facilitators

	**Undertake shared care**	**Undertake some aspects of shared care**	**Opt out of shared care**
	**Sheffield5patient, Sheffield7patient, Sheffield8patient, Sheffield9patient, York1patient**	**Sheffield6patient, York5patient, York6patient, York9patient, York12patient, York15patient**	**Sheffield3patient, Sheffield10 patient, Sheffield11patient, Sheffield20patient**
**Knowledge**	“we’ll say my potassium’s ok or my calcium’s alright, my Hb, my iron…we’ll compare like 'oh what’s yours like?’ **Sheffield7patient**	“they come in, get all the bits and pieces they need…they seem to cope alright with it so I take it does work” **York5patient**	“they just want to turn up and switch off” **York20patient**
	“you didn’t know what to do with it anyway so basically you just laid here and you didn’t know, you now thinking god what’s happening to me…then we started talking to each other…so we started asking questions with each other, and so it opened it up…whereas before you used to come and you’d say good morning, turn your telly on and go to sleep.” **Sheffield8patient**	“you’re not just laying there wondering what’s happening” **York5patient**	“everybody’s different and their views are different and perhaps how much information and processes they can manage at once” **Sheffield3patient**
	“some people think…you’ve got to do everything and we’ll say to them 'no you haven’t, you do what you want to do and what you’re capable of doing’…I don’t want to be forced into something what I don’t want to do…I’ve got no intentions in doing it at home you know” **Sheffield8patient**	“it’s quite empowering isn’t it to be able to do it you know, sort yourself out, you know and I’m not somebody who sits around you know” **York6patient**	“although I gave insulin to myself, because the needles were different you know and thinking am I gonna get it in” **Sheffield3patient**
		“all the other things about being on dialysis brings you know, osteoporosis and system not working properly, problems with bowels, sickness, cramp, you know there’s so many things that you have to deal with apart from having to come here three times a week” **York6patient**	“but with shared care there’s not going to be somebody there all the time is there?” **York20patient**
			“A unit’s opening up next door hopefully where we’ll be able to come and go more or less when we want, is that what it is?” **York20patient**
**Beliefs about capabilities**	“they don’t pressure you to do anything what you don’t want to do” **Sheffield5patient**	“I used to think gosh that’s good, I’d like to do that, to be independent” **York12patient**	“elderly patients…they’re frightened” **Sheffield3patient**
	“a sharp needle you feel it go into you, but once you’ve got this button hole done, the blunt needles just slip in…it’s like putting them into butter, you know it just slips in really, really good.” **York1patient**	“it makes me feel I’m in command, more responsibility” **York12patient**	“panicking…am I doing this wrong” **York20patient**
	“don’t know 3 to 6 months” **York1patient**	“there’s enough problems with the nurses doing it without you know the problems that I or anyone might cause to themselves” **York15patient**	“I just stopped… can’t be bothered anymore” **Sheffield10patient**
	“I’m a technophobe, and that’s like a computer to me you know…but once you get used to doing it and you have a sheet to follow.” **York1patient**	“when I saw you doing your needles I thought 'oh my god, am I going to have to do that?’…she says 'no, no, no’…and then a couple of month on, maybe because he knew he didn’t have to do it, he started doing it for his self” **York5patient**	
	“I’m always putting my long lifespan of my fistula down to me needling myself, I’ve always thought that’s got to be why, cause not many people get 16 years out of a fistula” **Sheffield7patient**	“a bad day when I come in and I just think 'just let me get on that chair and let myself go to sleep’” **York6patient**	
		“if someone’s 80 something you know you wouldn’t even go there” **York6patient**	
		“Hell of a lot to learn” **York6patient**	
**Skills**	“you know what to look for…if your venous pressure gets too high…it’ll set the alarm off..it could be a kink in the pipe…tape could’ve came undone…your needle might just need pulling out a little bit.” **Sheffield5patient**	“no respect to the nurses are doing different fistulas all day long aren’t they?… it doesn’t hurt when I put them in, there’s no pain whatsoever, I don’t feel anything, I know the tender spots and I know the bits that hurt” **York5patient**	“put needle in myself but I couldn’t do it” **Sheffield11patient**
	“I thought there’s no way I’m gonna even attempt it…they said about this button hole technique where you use a blunt needle…same track all the time, same site, obviously it scabs over, but it’s a bit like a pierced ear…just slide into it…they said you can’t bump it…it won’t go anywhere…I get on with it fine” **Sheffield5patient**	“I don’t like the idea of working with one hand you know like when you’re blood pressure with one arm…it’s frustrating” **York6patient**	“would like … but I can’t read or write” **Sheffield11patient**
	“she did the actual tunnelling, she made the button holes to begin with sharp needles…for a month…then she said 'right, now we’re going to put the blunt needles in’ she said 'and you’re going to do this yourself’, and I did and it was very easy.” **York1patient**	“I’d love to come in and line 10 machines…cause that’s how you learn…when you’re just doing one every so often you don’t remember…but you can’t do that cause of hygiene” **York6patient**	“she held it with me because I have these shakes sometimes, but that’s stopped me from doing it” **Sheffield11patient**
**Environmental context and resources**	“this year I went across…it was a private place…the Dr said to me 'no, I’m sorry we don’t do button holing’ so I said 'well I’m sorry I do’, because the nurses had given me all the needles and I was adamant I wasn’t going to lose these cause they were so good…he said on your head because of infections…the thing that amazed me was every morning…I had a crowd of nurses round me, they were all interested to see you know how it was done” **York1patient**	“I try to disassociate dialysis with my life and the less time I can spend here the better” **York5patient**	“not safe..if anything happens I would prefer to come to the hospital” **Sheffield11patient**
	Interviewer “before you got involved in the shared care, would you have looked at the folder at all?” Participant “no, no, I always thought that was purely what the nurses used” **York1patient**	“it was quite daunting walking in the first time…you don’t know what’s happening and everyone’s looking at you, get these needles out” **Sheffield6patient**	“you’ve got fixed times for your transport obviously, well you come in, a nurse puts you on the machine, your transport turns up and you go home, there’s no messing about with anything anymore” **Sheffield10patient**
	“I think I’d be more isolated at home” **Sheffield8patient**	“I think I’m past that, I just want it now, I want a kidney” **Sheffield6patient**	
	“because if something goes wrong I’ve got the nurses on hand, whereas if I was at home and something went wrong he would have to lift a phone or do something to the machine and I think he would panic, and lose his breath…so it’s better to come in” **York1patient**	“the idea is to reduce the numbers of nurses is it?” **Sheffield6patient**	
		“I wouldn’t want to spend that time setting that machine up” **York15patient**	
		“with all the messing about…so I’d had seven hours messing about with this one way or another, waiting about, hanging about, it’s not pleasant, it’s not nice” **York6patient**	

**Table 5:** Professional barriers and facilitators

	**Undertake shared care**	**Undertake some aspects of shared care**	**Opt out of shared care**
	**Sheffield1professsional, Sheffield2professional**	**Sheffield3professional, Sheffield4professional, York1professional, York4professional**	**York3professional**
**Knowledge**	“They didn’t know about their medication. They didn’t even know about their own care, their fluid restriction or anything… totally dependent on medical staff…here the patients are more educated. They’ve been given more about their care, their diseases…and advice, the treatment, why they’ve got renal failure. It’s a big difference here in their care.” **Sheffield1professional**	“sheer process of dialysis for the patient their ability to take in information” **Sheffield3professional**	
	“So they’re talking about their past, their non-dialysis days, what they’ve been doing, what they’ve been eating…we know more about them and the patient gets more from us…this is a two way combination” **Sheffield1professional**	“it’s a learning process for each individual constantly” **Sheffield3professional**	
	“they used to come to dialyse for 31/2 hours and go to sleep …now they’re discussing with each other…you will hear them discussing about their potassium, phosphates, Hb so they know more about themselves…they go online check their results, ask more questions.” **Sheffield1professional**	“patients want to learn more about their selves than they did before” **Sheffield4professional**	
	“I’ve had lots of things said…’it’s nothing to do with me, it’s not my fault, I come here, you dialyse me that’s your job, I don’t want to know’, right through to the 'I want to know everything about it, I want to know everything you’re doing and I want an explanation for everything you do’.” **Sheffield2professional**	“it must be awful, you’re not in control of what’s happening to you” **York4professional**	
		“it’s good for the staff as well to keep on track of things…cause everything changes all the time” **York1professional**	
		“we have an excellent renal service…anybody is encouraged to talk to anybody about anything…if you’ve got a concern about a patient and you want to ring a dietician, you ring the dietician” **York4professional**	
**Beliefs about capabilities**	“it was difficult to teach a patient who didn’t know anything about their own care” **Sheffield1professional**	“If they were more compliant and understand the process more” **Sheffield3professional**	“the little lady who thought it was more important that it looked neat than that is was clean” **York3professional**
	“I’m not a dietician…the dietician are always there, give them a ring and clarify, get the leaflet for the patient which gives me an idea to read it as well… improve my knowledge… if the senior nurses are there, the sisters are there, the ward managers are there, which have more experience…will answer my question as well which will be good for me.” **Sheffield1professional**	“we’ve had patients with learning disabilities that you would look at and thought “oh I don’t think they’d be up to doing this’ but have got on really well” **Sheffield3professional**	
	“the patient even knows …who will be the best to sort it out..they know about their condition, who to get and who not to get.” **Sheffield1professional**	“they’re more confident..they think oh I could do that and give it a try” **Sheffield4professional**	
	“a patient may challenge you but it’s also sometimes a request for information… so “well why am I doing this?’ and 'why do you want me to do that?’ may not be a challenge, it may actually just be a simple way of saying 'explain it to me a little bit further’.” **Sheffield2professional**	“there’s a fear…because it’s an electronic machine.. I reassure them there’s nothing that can go wrong.. it’s not going to damage anything…or blow up” **Sheffield4professional**	
		“they all say 'yeah it’s great’ they feel so much more confident and they feel in control” **York4professional**	
		“patients should have the choice” **York4professional**	
**Skills**	“so we show the staff are training as well as the patient” **Sheffield1professional**	“They’ve got phobias about needles” **Sheffield3professional**	“some people are just fine to come on and do whatever you tell them” **York3professional**
	It can be challenging “especially for the more junior members of staff who haven’t got the years of experience behind them” **Sheffield2professional**	“most of them have the ability and the skill to do a certain amount of it” **Sheffield3professional**	
		“they’ve got more control over what they’re doing” **Sheffield4professional**	“you need it even with the staff, cause you see people cut corners here and there” **York3professional**
		“you would have to put in place some sort of monitoring, revisiting, retraining, reassessment system, people do, we all you know, nurses slip back in habits don’t they” **York4professional**	
**Environmental context and resources**	“explain to the patient that it isn’t enforceable to go home…they were thinking that the NHS is going into crisis, they’re getting rid of the staff and most of the patients have to go home…their health will be in danger because the nurses won’t be there” **Sheffield1professional**	“weren’t encouraged to do anything for themselves” **Sheffield4professional**	“it was just a conveyer belt and all the patients basically had the same prescription and the same flows…it was just a real kind of industrial way of dialysing people, there was very little personalised care and you had no time to spend with them” **York3professional**
	“they’ll take them round to show, let the patient examine the machine, just sit down next to the patient, talk to the patient, you know take the anxiety out of it…Some of them haven’t seen the machine before.” **Sheffield1professional**	“it was very much you’re the patients, we’re the nurses, we look after you, we do it all for you” **York4professional**	“it’s really sort of embarrassing some days when you realise you can’t, you’re just doing the absolute bare minimum” **York3professional**
		“the prescription that we keep…some patients think that that’s ours…if it’s left on the table…they want to look at it…they can feel the difference if something’s dropped or something’s too high” **Sheffield4professional**	
	“before we bring the change for any equipment, like we introduced the staff training, the patient has been introduced to it as well.” **Sheffield1professional**	“there’s always somebody around if an alarm goes off…so you have to take a step back and wait for them to look …and they follow like the chain that we’ve said check this first and then that…and we’ll say 'oh what’s wrong with it?’ and they know, they can reel it off” **Sheffield4professional**	
	“We still run a production line dialysis unit and we still open at X and we still shut at Y, and the pressures are in actually finding time to allow people to take their time” **Sheffield2professional**	“spending anything over the allotted time with that patient was always difficult” **Sheffield3professional**	
	“Perhaps is where nursing sometimes gets itself wrong, you get to a senior position and you’re expected to sit in the office pushing pieces of paper about when in actual fact really, after 20 years experience perhaps we should be out on the ward, we’re passing on that knowledge and experience” **Sheffield1professional**	“there’ll always be patients that aren’t suitable to go home” **Sheffield3professional**	
		“the dialysis population is growing considerably all the time, so we’re always looking for ways to try and deal with that” **Sheffield3professional**	
		“the training process was cut down to 8 weeks from 6-12 months…it’s been really refined…to take them from shared care in the hospital to care at home it could be three weeks” **Sheffield3professional**	
		“I find that I’ve been there an hour and I was only scheduled for half an hour, but I don’t think, you’ve got to listen to what’s being said” **Sheffield3professional**	
		“It’s just the time and the staffing situation…sometimes it’s impossible to do it” **York1professional**	
		“it’s much harder for a main unit…because all, most of the acute work…they struggle with their daily workload…it’s harder for them to have a comprehensive programme or people to be dedicated towards spending time with patients” **York4professional**	
		“the chap that was months and months we just couldn’t leave him…if you’re stood there have an hour watching him try and put his needles in, your stood there…because he wanted to try” **York4professional**	

### Identification of barriers and facilitators to implementing SHC

Interviews were conducted with patients (n = 15) and professionals (n = 7) in five centres piloting SHC. A total of 26 patients were invited to participate; 19 responded, and 3 female patients (not engaged in SHC) chose not to participate. One female patient died before being interviewed. Patients varied in terms of age, gender, and level of disability - these specific characteristics are not presented to protect patient confidentiality; however, themes were consistent across participants. Nine professionals were invited, and two chose not to participate. All patients chose to be interviewed during haemodialysis [23], with the exception of one participant who was interviewed over the phone at home after transitioning to home dialysis. All professionals were interviewed in the haemodialysis centre where they worked.

Behavioural theory was used to identify key barriers (knowledge, beliefs about capabilities, skills and environmental context and resources). Data presented are primarily illustrative of the types of experience referred to by different categories of participant to illustrate the range of views from the relatively small number of participants within each category.

### How do patients experience SHC?

Benefits of participating in hospital-based SHC identified by patients included reduced waiting time, less pressure on family members or caregivers, isolation at home, increased knowledge of prescriptions, and improved fistula care. SHC patients reported that they now have more knowledge (of the dialysis procedure, their condition, dietary and fluid recommendations, and blood pressure); *e*.*g*., 'We’ll say my potassium’s ok or my calcium’s alright, my Hb, my iron…we’ll compare like 'oh what’s yours like’ (Knowledge Sheffield7patient).

A number of skills were learned by hospital-based SHC patients to manage the dialysis machine. Professionals provided progressive incremental skills training that assessed willingness and competency to perform goals set in collaboration with patients who expressed a desire to be involved in their own care (Beliefs about capabilities York1patient). Some patients progressed to self-needling using the button hole technique in which needles are passed down a subcutaneous track into the vein, even those that initially had a needle phobia (Skills Sheffield5patient). Others felt that they see the nurses having difficulty so don’t want to be involved in needling because of fear (Beliefs about capabilities York5patient). Those that were involved acknowledged that they could (and sometimes did) rely on professionals to take over in periods of ill health. These patients reported a sense of empowerment and control ('It makes me feel I’m in command, more responsibility’) (Beliefs about capabilitiesYork12patient). In centres that grouped SHC participants together, patients talked about the influence of older or less able patients acting as a motivating or enabling factor.

Barriers to participation included the belief that SHC meant independent self-care or having to do all of the competencies in preparation for a transfer to home haemodialysis or an unsupported unit staffed by limited numbers of professionals (Knowledge York20patient). Some patients perceived a risk of harm or fear at having to master complex machinery. There were also concerns about the safety of SHC, taking longer to dialyse, and missing patient transport. Those who opt out are thought to vary in the amount of information that they can process, and their willingness to get more involved (co-morbidities, impact of their diagnosis). Some patients are focusing on a kidney transplant in the short-term and don’t want to spend time learning new skills. Most patients thought that the folder containing their prescriptions was only to be viewed by nursing staff.

### How do professionals experience SHC?

Professionals perceived that they learned more about the patient and how they managed their condition; they could see improved outcomes (*e*.*g*., haemoglobin, potassium and phosphates); there was a reduction in time to train for home haemodialysis; and increased team working and benefits for patients who may have been excluded from home dialysis (*e*.*g*., elderly and learning disabilities).

Concerns were raised about patient safety, staff expertise to answer patient queries, and capacity to undertake SHC. A high standard of infection control including needle site sterilisation is required when haemodialysis is performed, and some professionals were concerned about infection rates increasing in patients who could lapse into bad habits ('the little lady who thought it was more important that it looked neat than that it was clean’) (Beliefs about capabilities York3professional). There was a concern that more junior members of staff would lack experience to answer complex patient queries. However, other staff commented that more junior healthcare assistants led the implementation of SHC, as they are more accustomed to answering questions and seeking support from other members of the clinical team. Some patients have none or limited knowledge of the condition and haemodialysis before starting. Several professionals likened this to training new nursing staff. In addition, haemodialysis requires ongoing education in management strategies as new technologies are introduced over time; one unit overcame this barrier by integrating teaching for patients and professionals at the same time. Time was identified as a constraining factor for a number of reasons. It is difficult to protect time to teach SHC while also having to manage acute patients. There were also time pressures involved in supporting patients who wished to participate but were not competent to manage independently without continuous supervision.

### Who needs to do what, differently, and what intervention components may overcome the identified barriers and enhance the facilitators?

Behavioural theory identified key barriers (knowledge, environmental context and resources), and enablers (beliefs about capabilities and skills). An intervention strategy that focuses on providing patients with information about the shared nature of care, how to access and read prescriptions and use machines; and professionals with skills to teach and continually review involvement of both professionals and patients may improve care.

### Shared learning event

Barrier and facilitator information was presented along with perceived benefits of SHC to a multidisciplinary group of stakeholders at a shared learning event. Participants supported that a culture of change involving patients and professionals was needed. Any intervention should include education and motivational techniques required to restore loss of confidence and increased responsibility or ownership of care that should be sustainable. They supported the call for evidence of cost effectiveness and patient inclusiveness, to confirm the evidence of benefits and enthusiasm from patient voices. Current patient-based evidence should contribute to influence care and policy making. Additional barriers of a lack of consistent terminology surrounding self/SHC and service redesign were acknowledged.

## Discussion

### Key findings

Coding unique experiences to behavioural theory allowed us to understand care from a staff and patient perspective. We have developed a tailored implementation intervention strategy that can be tested in a rigorous effectiveness evaluation to benefit patient care.

### Contribution to existing literature

Haemodialysis patients with insufficient knowledge have less control over their treatment and lives, and have a feeling of powerlessness [26]. Interventions that maintain patient control are important in long term conditions that often lead to a sense of helplessness [5] and a loss of control in social structures and environments beyond the dialysis unit [27], as they provide motivation to continue with lifesaving treatment [9]. Patient-centred care does not necessarily satisfy all patient demands [28]; instead, SHC aims to focus on the patients’ goals for behaviour change in parallel with the professionals’ duty to provide safe, efficient and equitable care [3].

Previous studies have identified lack of understanding, lack of self-efficacy, lack of medical supervision, and fear of social isolation as the most frequent barriers to peritoneal and home dialysis [22]. Our data go beyond individual barriers identified by patients to include barriers perceived to be important in implementing SHC from a professional perspective, incorporating individual and organisational barriers and facilitators.

It is thought that the organisation and structure of dialysis units impact on participation [27]. This was supported in our data. Settings that protected areas for those participating in SHC increased communication and support between patients. Units that dispersed those participating or not allowed other patients to view SHC and research has also shown that patients can act as mentors to other patients [29]. Our data supported this role.

Patient-centred care and autonomous care are difficult to implement, as they involve multiple stakeholders that may assume that it’s someone else’s responsibility [30], changing multiple behaviours. Healthcare delivery should be responsive to individual needs and preferences that go beyond simple checklists of behaviour [31]. Our study has attempted to build an intervention strategy to support a change in the culture of hospital-based dialysis from a passive patient, highly provider-directed service, to one of SHC as evidenced by the changes in patient communication and patient/professional partnership interactions. These data have implications for planning care [32] and policy.

### Strengths and limitations

Our in-depth exploration of barriers and facilitators to hospital-based SHC was conducted with a relatively modest sample of 22 interviews at 5 centres (2 hospitals and 3 satellite units). These centres were pragmatically chosen, as they were piloting the implementation of SHC in the UK. We purposively sampled patients and professionals who were undertaking some, all, or opting out of SHC to increase the diversity of our sample. The barriers and enablers identified in this study may not apply to patients who are not currently offered the opportunity to participate in SHC. In addition, these barriers and enablers may not apply to centres who are not involved in implementing SHC. We did not invite wider members of the team (*e*.*g*., dieticians, nephrologists, and social workers), as they were not involved in the day-to-day delivery of SHC. It was not possible to explore data saturation systematically [33] given the number of theoretical constructs included in our analyses; however, there was evidence of recurring themes as both patients and professionals described common barriers and enablers. We did not control for other factors that may affect participation, *e*.*g*., dialysis adequacy, disease severity, age, gender, knowledge, psychological distress, or levels of cognitive impairment. Participants in our study varied in terms of age, gender, and duration on haemodialysis, and two patients had returned to hospital-based haemodialysis following loss of a kidney transplant.

The interview schedule asked participants to talk about their personal experience of SHC. Experience data were constructed during the interview and may not represent reality, as they may be based on their reflections and what they were prepared to disclose. We chose to use the Theoretical Domains Framework to underpin our analyses, other behavioural theories do exist, and we feel that there are benefits from going beyond a single theory; to comprehensively examine individual and organisational barriers strengthened our study.

This study aimed to explore the impact of a patient-centred service; consequently, patients and carers were involved in all stages of the research (applying for funding, conducting the research, and reviewing the findings). The implementation researcher differed from the patient and caregiver researchers in her pre-understanding of dialysis. This may influence what was disclosed and what was probed further. The shared language and understanding of experience between participants and the patient/caregiver interview may have increased what was disclosed in the interviews. Patient interviews were conducted while the patient was on haemodialysis. This may have affected interview content, but minimised disruption to patients. Analyses of interview data suggested actionable messages are likely to be feasible (within existing resources) and acceptable (to patients and professionals); however, these messages may only be locally relevant. There was also an element of subjectivity involved in coding data, mapping barriers and facilitators, and exploring feasibility with stakeholders, which should be tested in a rigorous evaluation.

### Implications for research

There is a need to rigorously evaluate whether our multi-faceted intervention strategy to implement hospital-based SHC improves uptake, quality of life, and other healthcare outcomes. Interventions that increase knowledge and skills are thought to improve quality of life [34]. However, previous research has suggested that educational interventions are necessary but insufficient to increase self-care behaviours [6]. Our strategy includes a broader consideration of individual and organisational barriers to uptake that may improve implementation and outcomes. SHC was already being piloted in the centres included in this study, which may have already influenced some of the professional and organisational barriers to its implementation. The barriers identified in this study may not apply to other centres, or there may be additional barriers in centres that have not introduced SHC, and this should be explored in a further study.

Inter-rater reliability was not be explored statistically given the number of codes in the Theoretical Domains Framework. Other research teams have also noted difficulties in defining boundaries between domains [35], and this remains an area for further research to explore. Some text was coded to more than one construct [21]. We have not conducted a predictive theory-based survey [35] to identify the prevalence of these beliefs, as the aim of this study was to explore the range of barriers and facilitators identified by patients and professionals.

### Implications for practice

SHC should be discussed with all patients regardless of age or disability. The average age of haemodialysis patients has risen to 65 years [7] and is a frequent misconception that elderly patients are unable to participate in their own care [5]. These data illustrate that patients over 65 and with perceived disabilities are interested and do undertake SHC.

## Conclusions

Coding patient and professional experiences to the theoretical domains framework has allowed us to better understand the barriers to implementing SHC (*e*.*g*., knowledge, beliefs about capabilities, skills and environmental context and resources). Identifying barriers and facilitators has allowed us to develop a tailored intervention strategy most likely to optimise the implementation of SHC to benefit patients. Thus, we have produced an intervention that should be rigorously evaluated in a full scale randomised controlled trial.

## Competing interests

LG is an Editorial Board member of Implementation Science. All other authors declare that they have no competing interests.

## Authors’ contributions

LG, MW, SB and RC conceived the original idea for the study. LG, SB and KP collected data and conducted the first stage of analyses. LG and JH conducted the thematic analyses. LG drafted the manuscript, and all authors read and approved the final manuscript.
